# An international, cross-sectional comparative study of general practice curricula in medical school programmes

**DOI:** 10.5116/ijme.6a37.f280

**Published:** 2026-07-09

**Authors:** Rachel Roskvist, Shomel Gauznabi, Ellen Walker, Ranche Johnson, Kyle Eggleton, Susan Wells, Felicity Goodyear-Smith

**Affiliations:** 1 Department of General Practice & Primary Health Care, University of Auckland, New Zealand

**Keywords:** Medical students, medical schools, curriculum, general practice, family practice

## Abstract

**Objectives:**

To describe and compare how undergraduate general practice (GP) is taught across medical school programmes in selected high-income countries, to inform curriculum redesign at the University of Auckland, New Zealand.

**Methods:**

International observational cross sectional comparative study. Study participants were academic leads responsible for undergraduate general practice curricula in medical schools in Australia, England, Ireland, Canada, Norway, Iceland. Forty-four medical schools. Pragmatic purposive sampling to maximise diversity in geography, institutional age, programme structure, rural-urban context, student intake size. Single semi-structured interviews conducted April to November 2024, face-to-face or by videoconference. Interviews audio recorded with consent, transcribed verbatim, and supported by field notes. Data analysed using EPIC GP, modified SPICES derived framework examining electives, problem-based learning, curriculum integration, community based exposure, GP focused university teaching, and interprofessional education. Programmes coded and scored using a structured consensus process. Descriptive statistics and hierarchical cluster analysis used to identify patterns.

**Results:**

Marked variation observed in the organisation and visibility of general practice. Three curriculum typologies identified: hospital focused programmes with limited GP exposure; GP focused programmes with integrated, GP-led teaching; and community- or primary-health-care-focused programmes emphasising interprofessional education.

**Conclusions:**

This study provides an international comparative description of undergraduate GP teaching models. The findings demonstrate multiple approaches to embedding general practice beyond traditional block placements. Medical schools facing placement constraints may consider earlier GP engagement, increased GP led campus teaching, and selective use of simulation or digital learning. Further studies should examine educational and workforce outcomes.

## Introduction

A strong general practice (known in some countries as family medicine) workforce is the key to an effective, efficient and affordable health care system.^1^^,^^2^ However, there is a global shortage of general practitioners (GPs) to meet the health care needs of communities. The GP workforce crisis has been exacerbated by a number of factors: GPs retiring, early-career GPs opting for more flexible and reduced working hours, and insufficient medical graduates choosing a career in general practice.^3^ In addition, GPs are managing increasingly older, multi-morbid, complex chronic conditions in community settings. These factors can lead to low GP morale and burnout.

Across the Organisation for Economic Co-operation and Development (OECD), medical schools have been increasing their annual student intake and setting explicit targets for the proportion of medical graduates who pursue careers in general practice. This is often aspirational and not yet achieved. In the United Kingdom (UK) the expectation is for 50% to enter GP speciality training,^4^^,^^5^ although only 17% did so in 2015.^6^ Canada has a strategic goal of 40% of medical students selecting family medicine as their first choice.^7^ However, only 30% had this intention in 2011^8^ with no change in 2023.^9^ In 2024, more than six million Canadians are without a family physician.^10^ Australian medical graduates have increased their preference for a future GP career from 11% in 2011 to 19% in 2022, while in Aotearoa New Zealand (NZ), the proportion of graduates with general practice as the first choice for their future medical speciality dropped from 20% in 2016 to 14% in 2020.^11^

It is generally believed that increasing exposure to general practice during medical training will result in more graduates choosing this career. In 2015, only 13% of the UK medical education was delivered in primary care,^12^ leading Scotland to recommend that teaching in general practice should be 25% of the clinical curriculum.^13^ In response to the UK GP workforce crisis, the Wass Report of 2016 made a number of recommendations, including improved funding for medical student primary care training, widening participation selection criteria, increased access to quality experiences in general practice and multi-disciplinary teams in a variety of settings, exposure to positive GP role models across the programme, and a more integrated curriculum reflecting the patient journey through different health care settings.^14^ This has led to a number of changes in programmes, and to the opening of new medical schools with more integrated primary care-focused curricula.

There is some evidence that increasing the numbers of places for medical school entry for ethnic minorities, those from socially disadvantaged backgrounds or of rural origin positively impacts on subsequent choice to work in general practice in socially deprived or rural areas.^15^ Further, a literature review indicates that placements in underserved areas, including rural communities, especially in Australia, Canada and the United States, are beneficial for students, supervisors and the community.^16^

The Department of General Practice & Primary Health Care, University of Auckland has had a target of 50% of medical students becoming GPs since 2012.^17^ Most medical school general practice education occurs in the last three years of a six-year programme. Year 4 student attachments involve exposure to a wide range of health and social practitioners working in community and rural settings, whereas the focus for Year 5 and Year 6 students is authentic training and parallel consulting in urban and rural practices. Authentic experiences involve seeing real undifferentiated patients. However, there have been increasing shortages of general practice placement sites for undergraduate medical students hat is exacerbated by annual increases in the student intake, despite modelling and strategies to increase capacity.^18^ This is currently approximately a student intake of 320 per year, predicted to increase to over 350 per year over the next few years. Our department is currently reviewing and revising our undergraduate curriculum.

In the face of placement shortages and increasing student numbers, this study aimed to describe and compare how general practice is taught across medical programmes in selected high-income countries, to inform curriculum redesign at the University of Auckland, rather than to evaluate educational effectiveness or workforce outcomes.

## Methods

### Study design

This observational, cross-sectional comparative study using semi-structured interviews examined GP programmes delivered by different medical schools in six high-income countries.

### Participating universities and key informants

Australia, Canada, England, Ireland, Norway and Iceland were targeted as countries having similar health systems and medical programmes to that of New Zealand. Approach to key informants in departments of general practice for interviews was pragmatic, based on our existing knowledge of, and sometimes established contacts in, the institution and the populations they serve. We obtained contact details for key participants, and wherever practical, we conducted in-person visits. Medical schools were selected for maximum diversity, rural versus urban, and traditional programmes versus newly established schools.

Teaching heads in the relevant general practice departments were identified by personal connections and networks of the researchers, or by online searches, and invited to participate by email. Contacted participants were asked to pass the email to colleagues if they were inappropriately identified as the lead academic. Participant Information Sheets were provided. Informed consent was obtained orally, recorded and transcribed. While key informants were not anonymous to the interviewing researcher, they were not identified in the database, and the universities were assigned numerical codes. However, given the specific characteristics of some of the programmes, it is possible that some of the universities and the teaching heads may be identifiable. No personal or sensitive information about the key informants was obtained.

Ethical approval was obtained from the University of Auckland Human Participants Ethics Committee on 6/05/2024 for three years.

### Interviews

Appropriate key people were interviewed from 44 universities. Participants underwent single semi-structured interviews about the structure and nature of their programmes conducted by three experienced GP academics (FG, RR, SG) and one nurse practitioner (EW) between April and November 2024. These were face-to-face wherever possible, otherwise via zoom. Contemporaneous notes were taken, interviews audiotaped with oral consent recorded, audio-files AI-transcribed and data entered into a spreadsheet.

### Theoretical framework

Data were initially examined using the SPICES framework described by Harden which assists with understanding the educational strategies used in medical education and when considering curriculum (re)design.^19^ The SPICES framework components are Student-centred versus teacher-centred; Problem-based versus information gathering; Integrated versus discipline-based; Community-based versus hospital-based; Electives versus standard programme, and Systematic versus apprenticeship-based or opportunistic. The SPICES framework has been used extensively in medical education to examine where curricula sit along the six educational continua and has strong face and construct validity as an educational framework. SPICES is considered methodologically appropriate for descriptive and comparative studies, but it is not a validated measurement instrument.

We selected this framework to enable us to standardise, audit and compare against elements of curriculum design. Ultimately, we had sparce data identifying student or teacher-centred features. Further, the systematic approach of SPICES relies on students keeping a logbook against a required list of patients with specific diseases seen and skills mastered. While this may be appropriate in medical speciality training in hospitals, in general practice patients present with undifferentiated concerns, complaints and symptoms, not diagnoses. The key element for students to learn is the broad scope of patient care in the context of people’s lives, rather than diagnosis and treatment of specific diseases, and hence for this discipline, the opportunistic model of seeing ‘whomever walks through the door’ is more suitable than the systematic approach.

**Figure 1:**
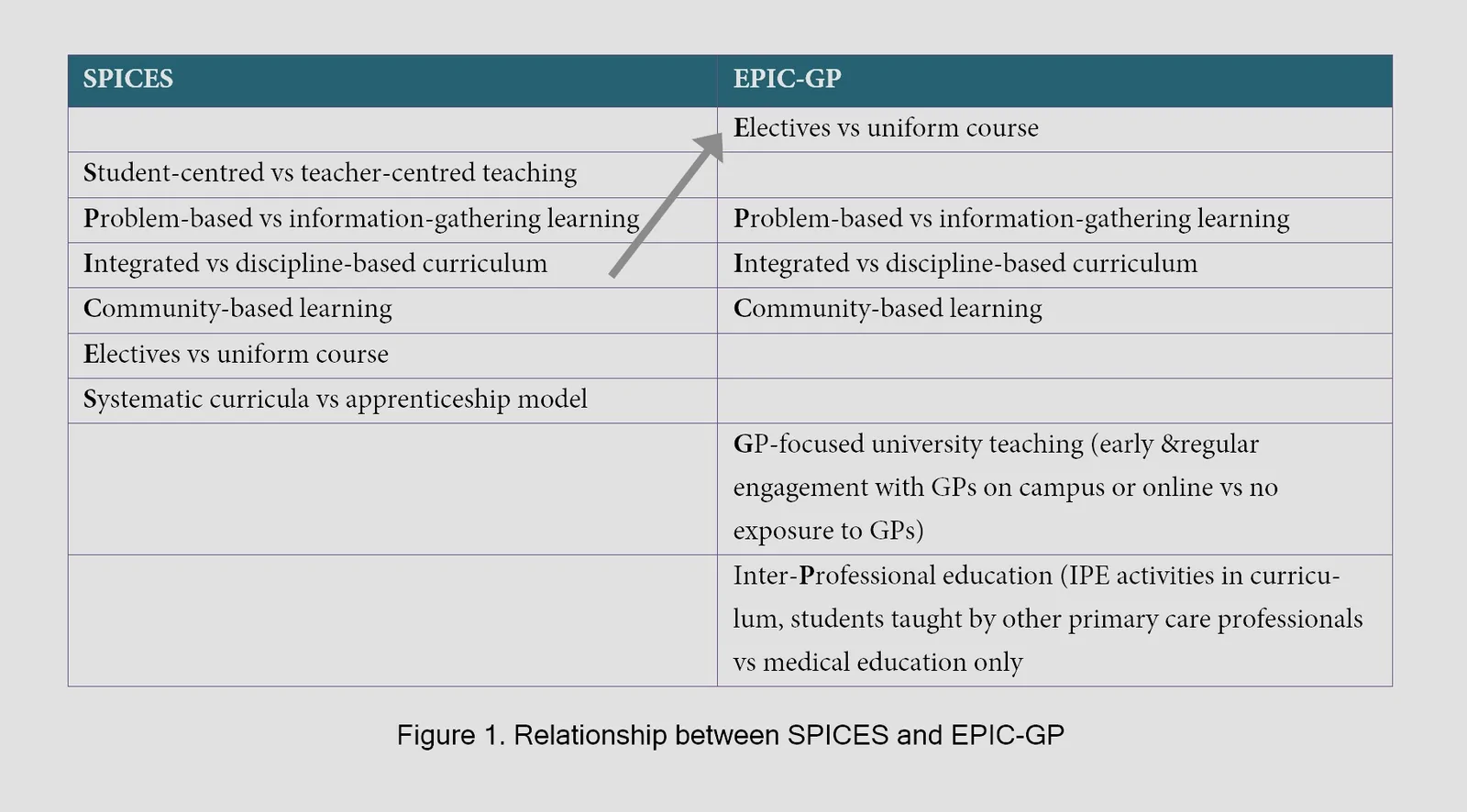
Relationship between SPICES and EPIC-GP

We therefore created a modified SPICES framework called EPIC-GP, whereby Electives (additional GP exposure) versus standard curriculum programme, Problem-based versus information-gathering, Integrated versus discipline-based, and Community-based versus hospital-based categories remain, but Student-centred versus teacher-centred and Systematic versus apprenticeship-based or opportunistic are replaced with GP-focused university-teaching (students have early and regular engagement with GPs on campus or online versus no exposure to GPs or information given on campus is from the GP perspective) and Professional education (interprofessional education (IPE) activities within the curriculum or students taught by other primary care professionals, versus taught by doctors only) – Figure 1. In short, we adapted SPICES to better reflect the educational context of general practice, and our resulting EPIC-GP framework was used to support systematic comparison.

### Nature of data

Data collected from the semi-structured interviews included the date when the medical school was established, nature of the medical programmes including undergraduate or graduate, student intake numbers and details of the GP component in the curriculum, as well as the challenges, innovative aspects, solutions developed, and perspectives from key informants about what has worked/been successful, and what has not (Supplement 1). Qualitative findings are to be published elsewhere.

### Analyses

Interview transcripts and contemporaneous notes were reviewed in full and analysed using the EPIC GP framework. One researcher (RJ) undertook the primary coding by systematically mapping interview data to each EPIC GP domain (Electives, Problem based learning, Integrated curriculum, Community based exposure, GP focused university teaching, and Professional/ interprofessional education). A second researcher (FG) provided iterative review, feedback, and interpretive oversight throughout the analytic process.

For each domain, evidence from the interviews was synthesised to assign an overall score on a five-point ordinal scale, based on the extent to which the programme demonstrated the characteristics defined in the EPIC GP matrix. Scoring was undertaken iteratively, with provisional scores refined through repeated reference to the source data. The final coding decisions and domain scores were reviewed and discussed with FG and discrepancies or uncertainties were resolved through interpretive discussion to achieve conceptual consensus.

EPIC-GP scores were summed and correlation statistics calculated between EPIC-GP variables, type of programme and year of establishment using Stata 19. Cluster analysis was then undertaken on the data. Correlation and cluster analyses were undertaken as exploratory, descriptive tools to identify patterns and potential typologies across programmes, rather than to test predefined hypotheses.

A hierarchical cluster analysis using Ward’s minimum linkage variance method in Stata 19 was performed and a cluster dendrogram generated to determine an appropriate number of clusters. Descriptive statistics were used to describe the three clusters and subsequent proposed typologies of medical schools. Factor analysis on EPIC-GP was not carried out due to the underpowered sample.

## Results

### Description of sample

Our pragmatic sample consists of 44 medical schools in the high-income countries of Australia, Canada, England, Ireland, Norway and Iceland. Fifty-seven were approached: ten did not reply, one declined and two were unable to schedule a suitable time. The programmes range from very long-standing to recently established programmes, with annual student intakes ranging from <50 to >400 (Table 1) and from traditional lecture-based preclinical followed by clinical years, through to innovative integrated, problem-based, or spiral curricula.

### EPIC-GP scores

**Figure 2:**
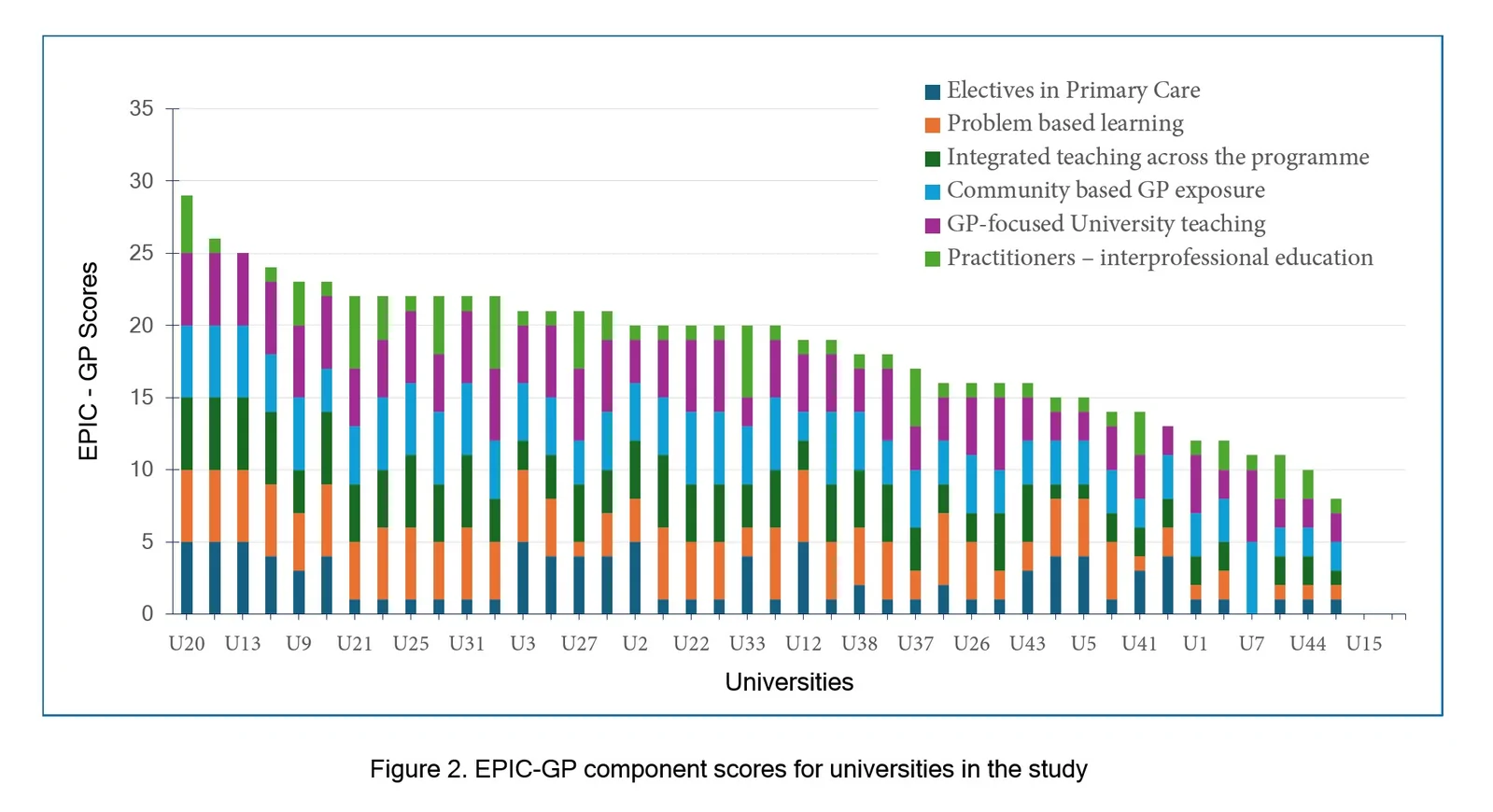
EPIC-GP component scores for universities in the study

The EPIC-GP matrix with examples of high and low scores from responses are shown in Table 2. The range in EPIC-GP scores for each university can be seen in Figure 2.

### Correlations

Correlation analysis shows moderately strong correlation between integrated and GP-focused university teaching, and moderate correlation between integrated teaching and community-based GP exposure and attachments, between problem-based learning and community-based attachments, and between GP-focused university teaching and community-based attachments. There is weaker correlation between problem based learning and integrated teaching, and problem-based learning and GP-focused university teaching. Elective and other practitioners’ teaching are not correlated with other variables (Appendix Table A). There is little to no correlation with whether the medical programme is graduate or undergraduate or the year the medical school was established.

### Clusters

**Figure 3:**
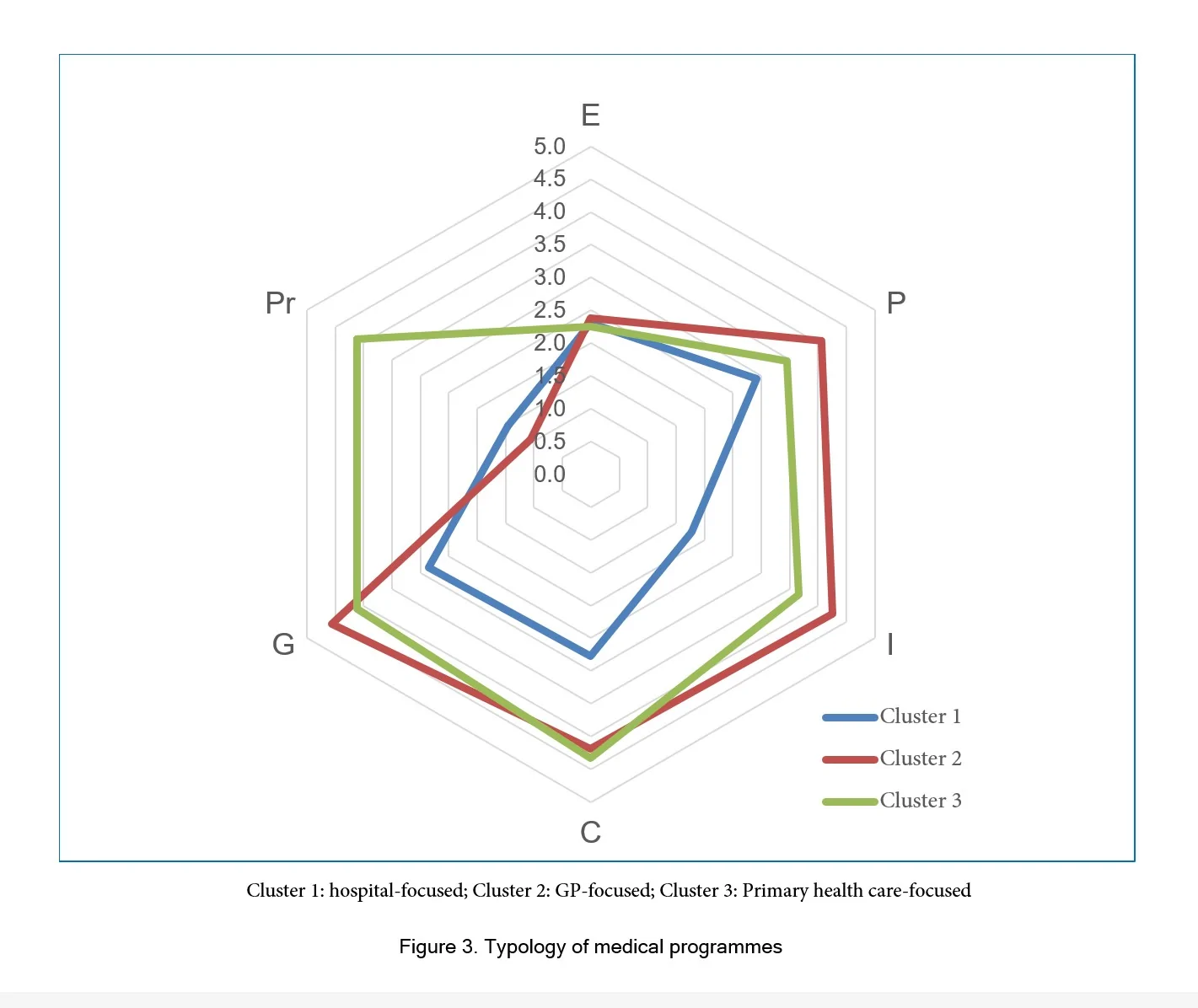
Typology of medical programmes

Exploratory cluster analysis suggested three potential typologies of medical school approaches to general practice teaching (see Figure 3 and Appendix Table B). The first cluster is medical schools with low-medium scores across the majority of EPIC-GP variables, which we designate hospital-focused teaching and learning programmes. The second cluster is medical schools with high scores across the majority of EPIC-GP variables with the exception of other practitioners teaching and electives, designated GP-focused programmes. The third cluster is medical schools with medium to high scores across the majority of EPIC-GP variables, including teaching and learning with and by other health professionals, but with the exception of electives. We designate these ‘community- or primary health care-focused programmes’.

**Table 1:** Characteristics of the 44 medical schools

Characteristic	N	(%)
Country		
Australia	16	(36)
Canada	4	(11)
England	15	(33)
Ireland	6	(14)
Nordic	3	(6)
Type of programme		
Undergraduate entry	21	(48)
Graduate entry	13	(30)
Both	10	(22)
Age of university / programme		
200-300+ years (1711 to 1825)	4	(9)
100-199 years (1826 to 1925)	9	(21)
50-99 years (1926 to 1975)	9	(21)
25-49 years (1976 to 2000)	5	(11)
10 to 24 years (2001 to 2015)	12	(27)
1 to 9 years (2016 to 2025)	5	(11)
Length of the programme		
3 years	1	(2)
4 years	13	(30)
5 years	14	(32)
6 years	11	(25)
Varies with type of programme	5	(11)
Student intake numbers		
<50	2	(4)
50 to 99	6	(14)
100 to 149	7	(16)
150 to 199	6	(14)
200 to 249	12	(27)
250 to 299	1	(2)
300 to 349	4	(9)
350 to 400	3	(7)
>400	3	(7)

## Discussion

We found a moderately strong correlation between four variables – problem-based learning, integrated curriculum, community-based and GP-focused university teaching. These variables appeared to account for the majority of variation between medical schools. The commonality between these variables is centring general practice within the teaching and learning environment. There is little to no correlation regarding whether the programme was undergraduate or postgraduate, nor whether the medical school was long or recently established.

**Table 2:** EPIC-GP coding matrix with examples

**Score**	**E**	**P**	**I**	**P**	**G**	**Pr**
**5**	**Elective**Selected students can choose to study GP in depth throughout programme; to have additional GP or other primary health care attachments.Special rural or GP stream in programme	**Problem-based learning**Complex real-world problems used to promote student learning of concepts and principles from onset of programme	**Integrated teaching**Disciplines taught in context of general practice e.g. body systems on campus taught through GP lens; longitudinal integrated clerkship (LIC); rural immersion programmes	**Community-based (GP) exposure & attachments **Much of teaching takes place in general practice, rural hospital or other primary care settings with students regularly consulting with patients; students start GP attachments in Year 1; GP attachments in several years.Students do home visits.Students work in or visit communityservices	**GP-focused university-teaching**Students have early & regular engagement with GPs on campus or onlineSmall-group GP tutorsGPs teach communication and clinical skillsStudents see selected general practice or standardised / simulated patients to learn relevant basic sciences and clinical manifestations of conditions	**Practitioners**Curriculum includes interprofessional educational (IPE) activities; students taught by other primary care professionals as well as GPs
Examples	18/52 elective with family medicine encouraged. Optional resources available to study aspects in greater depth. Student can elect to be in special rural or GP stream	Curriculum based on problem-based learning with small-group case-based teaching on campus.Cases developed by teachers and/or students.Cases presented to peers via small groups.Few or no lectures – focus on interactive teaching. Simulation and interprofessional scenario work.	Clinical teaching starts at beginning of programme - no pre-clinical then clinical years. Teachers provide clinical context and cases to integrate bio-medical concepts. LIC for 1 year in general practice and rural medicine.	GP attachment in every year.Early patient contact & family attachment schemePairs of students visit GP-selected patients with long-term conditions 3-4 times in home over 4 months.Learning opportunities in primary care / community settings eg drug & alcohol, NGOs, homeless shelters, migrant centres, hospiceLongitudinal well child development module	GPs teach history-taking, clinical examination, population health, disease-specific content, social determinants of health, inequities. Students have GP tutors for the year.May involve discussion forums.Students taught clinical and communication skills by GPsGPs teach anatomy, physiology, biochemistry, pathology using selected patientsGP teach large proportion of curriculumGPs are leads for Yrs 3 & 4	I IPE with midwives, PA, nurse, paramedic - simulation of advanced skillsYrs 3, 4 5 alternate GP clinic andwider PC team (eg nurse, care practitioners, physio, pharmacists, social prescribers)Community & creative health includes nurses, midwives, PA, dietetics, women & child health
**1**	**Standard programme GP**All students pass through set of prescribed courses with no opportunity to study subject of own choosing or in more depth	**Information gathering**Information didactically imparted to students on large body of facts on basic science and clinical knowledge	**Discipline based**Each discipline teaches separatelyPre-clinical disciplines eg anatomy, physiology taught first then clinical disciplines including general practice subsequently.	**Hospital-based**Teaching centred around main teaching hospital. No GP visits or attachments and no exposure to other community-based health and social services	**Specialist-focused campus teaching**None of the clinical information given on campus eg in lectures is from the GP perspective	**Medical teaching only**Students taught medicine with no IPE and no teaching by non-medical practitioners or with non-medical students
Examples	No opportunity to study general practice other than through the standard programme	Traditional programme with system organ blocks such as cardiology, respiratory etc.	Preclinical & clinical years. Basic sciences taught in 1^st^ years followed by clinical (clerkship) years	Few or no GP attachmentsNo visits or exposures to homesNo visits to community-based services	No GP teaching on campus across programme.No teaching of conditions from GP perspective	No IPE activities. No teaching with or by other professions

NB: Score 4: Programme has most of features; Score 3: Programme has some of features; Score 2: Programme has one of features; Score 1: Programme has none of features

Our study describes substantial variation in how medical schools structure undergraduate exposure to general practice, with many programmes emphasising community based and authentic clinical experiences. While the present study did not examine student career intentions or workforce outcomes, these observed approaches align with a wider body of literature exploring the relationship between undergraduate general practice exposure and subsequent career choice. There is evidence indicating that the quality and duration of undergraduate GP experience is associated with increased likelihood of choosing a GP career,^20^^-^^24^ although most studies have weak methodologies and potential biases.^25^

Our study highlighted a strong focus on community-based experiences. A study of 29 English universities found that the quantity of authentic general practice teaching (teaching in a practice with patient contact, in contrast to non-clinical sessions such as group tutorials on campus) at each medical school was significantly positively associated with the percentage of its graduates who entered general practice training in both 2014 and 2015.^26^ Focus groups with UK medical students indicated early, high-quality, ongoing and authentic general practice exposure led to an increased intent to pursue a GP career.^27^

However, the relationship between undergraduate experience and career choice is neither consistent nor straightforward. In Ireland, the proportion of graduates entering GP training varied widely across medical schools and was not correlated with time spent in general practice placements.^28^ A UK survey of applicants to GP training found that positive undergraduate GP experiences had only a modest direct association with speciality choice,^29^ and New Zealand data similarly indicate that undergraduate experience ranks relatively low among factors influencing career decisions.^11^ Limited evidence also suggests that graduate entry programmes produce more GPs.^30^ These findings underscore the complexity of GP career choice and caution against assuming direct causal links.

Within this broader context, our study identified that some medical schools offer longitudinal integrated clerkships (LICs) or extended general practice placements for selected students. LICs are characterised by students participating in the provision of comprehensive care of patients over time, with ongoing learning relationships with their supervisors, and meeting the majority of the year’s core clinical competencies across multiple disciplines simultaneously in this context.^31^ LICs have been established internationally in both rural and urban contexts. There is some evidence that students who experience a rural LIC are more likely to make primary care and rural career choices.^32^^,^^33^ The Irish medical school with an 18-week LIC has found that 43% of alumni are working in general practice six to eight years following graduation.34 A German study found a four-month GP clinical rotation during the final year was associated with an increased choice of GP as a career.^35^

Interprofessional teaching accounted for the majority of the remaining variation in the EPIC-GP cluster analysis, with one particular cluster of medical schools including interprofessional teaching within their GP curriculum. Primary care is increasingly delivered by interprofessional teams; hence exposure to other healthcare disciplines and IPE is increasingly seen as an important aspect of undergraduate medical education, although formal evidence of its effectiveness is sparce.^36^

Offering electives in general practice did not feature strongly in the cluster analysis or in the thematic analysis for proposals to increase general practice exposure. This is not surprising considering that electives may place increasing pressure on GP departments to find placements additional to regular attachments. Further, a systematic review found mixed evidence.^37^ One study found no association between elective experience in resource-poor settings and a preference for primary care or rural practice,^38^ although another found a positive association with a pre-clinical GP elective.^35^

While this study did not evaluate student attitudes, career intentions, or workforce outcomes, the patterns observed in programme structure can be interpreted in light of existing literature examining associations between undergraduate general practice exposure and subsequent career choice. Traditionally, medical schools did not recognise general practice as an academic discipline, and did little to promote it as a career. The world’s first professor of general practice was appointed at the University of Edinburgh in 1963, and departments were subsequently founded at universities in England, Norway, Canada and the Netherlands.^39^ In many countries, such as Australia and New Zealand, departments were not established until the 1980s. Many countries have experienced lower-than-desirable numbers of medical students training to be GPs. One of the factors contributing to this has been identified as professional negativism where secondary specialities perceive primary care as lower status. The Wass Report in England, mirrored by initiatives in other countries, recommended that medical school curricula offer a more integrated less speciality organised approach, reflecting the patient journey including across the primary-secondary care interface.^14^ The report recommended that GP role models should be visible across the entire programme, with increased general practice placements.

This has seen the introduction of case-based learning, integrated and spiral curricula, GPs teaching much of the early curriculum and increased community placements in many of the English medical schools in our study. The strong correlation observed between integrated curricula and GP focused university teaching is therefore perhaps unsurprising. GPs with their generalist and holistic approach are best-placed to deliver content across disciplinary boundaries.

Despite being exploratory in nature, we believe that our three different typologies based on low, medium-high and high EPIC-GP scores - hospital, GP or community/primary health care-focused are important. Curricula with early regular exposure to GPs as role models and authentic experiences in general practices are likely to increase preference for a GP career. However, the complexity of looking after aging populations with multiple chronic conditions has helped shape general practice as a team activity. It is anticipated that students taught alongside other primary care providers such as nurses and pharmacists in IPE programmes, and by other health professionals in community-based settings will develop deeper understanding of the different roles and the team dynamics required for primary care provision.

### Strengths and limitations

This study has a number of strengths. It draws on a large and diverse international sample, incorporating perspectives from leaders of general practice curricula at 44 medical schools across six high-income countries. Access to senior academic informants enabled insight into programme-level curriculum design, strategic priorities, and educational constraints rather than individual teaching experiences. The comparative cross-national approach allowed identification of common patterns and innovations across differing institutional and national contexts. Use of semi-structured interviews provided rich descriptive data, while the systematic and team-based analytic approaches enhanced credibility and transparency.

However, there are several limitations. Medical schools were selected through pragmatic sampling, which may have favoured institutions with existing engagement in curriculum innovation or stronger academic general practice profiles. Data were based on self-reported programme descriptions rather than direct observation or documentary analysis, introducing potential reporting bias. As a cross-sectional study, findings represent a snapshot in time and do not capture curriculum evolution nor longitudinal outcomes. The study did not examine student learning outcomes nor career decisions, limiting conclusions about the effectiveness of identified approaches. Finally, because the sample was restricted to high-income countries with broadly comparable health systems, transferability to other contexts may be limited.

This study did not assess student career intentions, workforce outcomes, or educational effectiveness, and therefore cannot establish links between programme design and subsequent career choice. Our observations related to potential workforce implications are therefore interpretive and informed by existing literature rather than the primary data.

### Implications

This study provides a descriptive, international comparison of how undergraduate general practice curricula are structured and delivered across medical schools in high-income countries. The findings demonstrate that there is no single dominant model for integrating general practice within medical programmes; instead, institutions employ a range of approaches that balance practice-based learning, campus-based teaching, simulation, longitudinal experiences, and digital modalities. For curriculum planners, these findings offer a menu of feasible strategies that have been adopted in comparable contexts facing similar placement capacity constraints.

For the University of Auckland, the study has practical implications for curriculum redesign. The international examples identified in this study have directly informed our local consideration of how general practice exposure can be redistributed across the programme, including earlier engagement with general practice, greater use of GP-led campus teaching, and selective replacement of in-practice time with simulation and digital learning where appropriate. Importantly, the study supports the view that meaningful exposure to general practice does not rely solely on traditional block placements, which may be increasingly unsustainable.

More broadly, the findings highlight the importance of making general practice visible as an academic discipline within medical schools. Many programmes described embedding GPs as teachers across clinical, communication and case-based learning, suggesting that the way general practice is positioned within the formal and hidden curriculum warrants careful consideration by medical educators.

## Conclusion

This study provides an international descriptive comparison of how undergraduate general practice curricula are organised and delivered. It highlights a range of structural approaches offering comparative insights that may inform local curriculum redesign. However, further research is required to examine educational and workforce outcomes.

### Acknowledgements

Thanks to all the educational leaders who freely gave their time to be interviewed about their medical programmes. This work was supported by the John and Jill Richards Professorship Fund under Grant number MAL 020/24.

### Conflict of Interest

The author**s** declare that there is no conflict of interest.
