# Supplementary material for: An international, cross-sectional comparative study of general practice curricula in medical school programmes
Source: Int J Med Educ. 2026 Jul 9;17:109–18. doi: 10.5116/ijme.6a37.f280 (PMC13563277; doi:10.5116/ijme.6a37.f280)
Supplement: Supplementary file 1 [file ijme-17-109-S1.pdf]

Appendix

Table A. Correlation statistics for EPIC-GP

	E	P	I	C	G	Pr
E	1					
P	0.17	1				
I	0.01	0.434	1			
C	-0.08	0.60	0.65	1		
G	0.06	0.49	0.71	0.55	1	
Pr	-0.045	-0.27	0.048	0.07	-0.08	1

Table B. Mean score for each cluster

Cluster	E	P	I	C	G	Pr
1	2.3	2.9	1.8	2.8	2.8	1.5
2	2.4	4.1	4.3	4.2	4.6	1.1
3	2.3	3.4	3.7	4.3	4.1	4.1
